# Prognostic Value of Combing Primary Tumor and Nodal Glycolytic–Volumetric Parameters of ^18^F-FDG PET in Patients with Non-Small Cell Lung Cancer and Regional Lymph Node Metastasis

**DOI:** 10.3390/diagnostics11061065

**Published:** 2021-06-09

**Authors:** Yu-Hung Chen, Sung-Chao Chu, Ling-Yi Wang, Tso-Fu Wang, Kun-Han Lue, Chih-Bin Lin, Bee-Song Chang, Dai-Wei Liu, Shu-Hsin Liu, Sheng-Chieh Chan

**Affiliations:** 1 Department of Nuclear Medicine, Hualien Tzu Chi Hospital, Buddhist Tzu Chi Medical Foundation, Hualien 97002, Taiwan; jedimasterchen@hotmail.com (Y.-H.C.); kaopectin@yahoo.com.tw (S.-H.L.); 2 School of Medicine, College of Medicine, Tzu Chi University, Hualien 97002, Taiwan; oldguy-chu1129@umail.hinet.net (S.-C.C.); tfwang@tzuchi.com.tw (T.-F.W.); ferlin@tzuchi.com.tw (C.-B.L.); dwliu5177@yahoo.com.tw (D.-W.L.); 3 Department of Hematology and Oncology, Hualien Tzu Chi Hospital, Buddhist Tzu Chi Medical Foundation, Hualien 97002, Taiwan; 4 Epidemiology and Biostatistics Consulting Center, Department of Medical Research, Hualien Tzu Chi Hospital, Buddhist Tzu Chi Medical Foundation and Department of Pharmacy, School of Medicine, Tzu Chi University, Hualien 97002, Taiwan; wangly1212@gmail.com; 5 Department of Medical Imaging and Radiological Sciences, Tzu Chi University of Science and Technology, Hualien 97005, Taiwan; john.lue@protonmail.com; 6 Department of Internal Medicine, Hualien Tzu Chi Hospital, Buddhist Tzu Chi Medical Foundation, Hualien 97002, Taiwan; 7 Department of Cardiothoracic Surgery, Hualien Tzu Chi Hospital, Buddhist Tzu Chi Medical Foundation, Hualien 97002, Taiwan; rr122336@gmail.com; 8 Department of Radiation Oncology, Hualien Tzu Chi Hospital, Buddhist Tzu Chi Medical Foundation, Hualien 97002, Taiwan

**Keywords:** ^18^F-FDG, PET, non-small cell lung cancer, prognosis, glycolytic, volumetric

## Abstract

We investigated whether the combination of primary tumor and nodal ^18^F-FDG PET parameters predict survival outcomes in patients with nodal metastatic non-small cell lung cancer (NSCLC) without distant metastasis. We retrospectively extracted pre-treatment ^18^F-FDG PET parameters from 89 nodal-positive NSCLC patients (stage IIB–IIIC). The Cox proportional hazard model was used to identify independent prognosticators of overall survival (OS) and progression-free survival (PFS). We devised survival stratification models based on the independent prognosticators and compared the model to the American Joint Committee on Cancer (AJCC) staging system using Harrell’s concordance index (c-index). Our results demonstrated that total TLG (the combination of primary tumor and nodal total lesion glycolysis) and age were independent risk factors for unfavorable OS (*p* < 0.001 and *p* = 0.001) and PFS (both *p* < 0.001), while the Eastern Cooperative Oncology Group scale independently predicted poor OS (*p* = 0.022). Our models based on the independent prognosticators outperformed the AJCC staging system (c-index = 0.732 versus 0.544 for OS and c-index = 0.672 versus 0.521 for PFS, both *p* < 0.001). Our results indicate that incorporating total TLG with clinical factors may refine risk stratification in nodal metastatic NSCLC patients and may facilitate tailored therapeutic strategies in this patient group.

## 1. Introduction

The incidence of lung cancer is the highest among all types of cancers, with lung cancer being the leading cause of cancer mortality worldwide [1,2,3]. Non-small cell lung cancer (NSCLC) accounts for 85% of all lung cancer cases [4,5,6]. In patients with NSCLC without distant metastasis, regional lymph node metastasis is common; 13.0% to 40.3% of these cases develop nodal metastases despite early primary tumor status [7]. With current therapeutic advances, regional nodal metastasis without distant spreading can be curatively treated by definitive concurrent chemoradiotherapy (CCRT), radiotherapy, or surgery. However, the treatment response and survival outcome of NSCLC cases with regional nodal metastasis are quite heterogeneous. The 5 year overall survival (OS) rates range from 9% to 60% [8,9,10,11]. Furthermore, the nodal classification in the eighth edition of the American Joint Committee on Cancer (AJCC) may be inadequate for prognostic stratification of NSCLC cases with regional lymph node metastasis [11,12,13]. Therefore, a more reliable prognosticator is imperative in this patient group to guide more sophisticated risk-adapted treatment strategies.

^18^F-fluorodeoxyglucose (^18^F-FDG) positron emission tomography (PET) is highly sensitive for detecting the disease extent of NSCLC, and this imaging modality has become the standard-of-care tool for staging and re-staging patients with NSCLC [14]. Because ^18^F-FDG PET provides a way of featuring the glycolytic activity of the tumor, it is also able to represent the tumor viability and can be used to assess the treatment response [15]. Furthermore, the glycolytic activity in tumors is associated with vicious signaling pathways [16,17]. Several ^18^F-FDG PET semiquantitative parameters have been developed to quantify the glycolytic activity and metabolic volume of tumors, including standardized uptake value (SUV) and volumetric parameters such as the metabolic tumor volume (MTV) and total lesion glycolysis (TLG). Higher metabolic activity and larger metabolic burdens are associated with worse survival outcomes; thus, many studies have focused on the use of ^18^F-FDG PET-derived semiquantitative parameters as prognostic biomarkers to predict survival outcomes in patients with NSCLC [15,18,19,20,21]. In addition, the semiquantitative ^18^F-FDG PET parameters can not only be derived from the primary tumor but can also be measured from the metastatic nodes, as the genotypes and the consequent phenotypes may not be the same in the metastatic lesions and primary tumors [22,23]. To date, most studies for nodal metastatic NSCLC have evaluated the ^18^F-FDG PET parameters from the primary tumor and metastatic lesions separately; studies combining ^18^F-FDG PET parameters from both metastatic nodes and primary tumor in nodal metastatic NSCLC are limited [21].

Therefore, the objective of this study was to investigate the feasibility of combining ^18^F-FDG PET parameters from primary tumors with regional metastatic nodes to assess the survival outcomes in patients with nodal-positive NSCLC without distant metastasis.

## 2. Materials and Methods

### 2.1. Patient Population

This retrospective study was conducted in accordance with the Declaration of Helsinki and the protocol was approved by the local Institutional Review Board and Ethics Committee at our hospital (IRB109-235-B). Due to the retrospective nature of this study, the requirement of informed consent for this study was waived. We retrospectively enrolled patients with a new diagnosis of NSCLC from January 2010 to September 2019. The diagnoses of NSCLC in all study patients were established using histopathology. Serial examinations were performed in all study participants for lung cancer staging and treatment planning at the time of the initial diagnosis. The examinations included contrast-enhanced computed tomography (CT) of the chest to the upper abdomen, ^18^F-FDG PET/CT, gadolinium-enhanced MRI of the brain, and pulmonary function tests. For lesions in the images that were indicative of malignancy, image-guided biopsies were collected whenever possible. If biopsies were not feasible or if the biopsy result was negative, the patient was closely monitored with imaging. Patients were re-staging according to the eighth edition of the AJCC staging manual [13]. We only included patients with a clinically positive regional nodal metastatic disease (patients with an N1 to N3 classification were included) and without evidence of distant metastasis at the time of the initial diagnosis. Patients’ daily living performance at the initial diagnosis was assessed using the Eastern Cooperative Oncology Group scale (ECOG) [24]. Patients included in this study received curative surgery (resection of the primary tumor and mediastinal lymph node dissection) with or without neoadjuvant CCRT, definitive CCRT, or definitive radiotherapy as the initial treatment. The radiotherapy dose was 2 Gy/fraction daily up to a targeted dose of 60 to 66 Gy. A cisplatin-based chemotherapeutic regimen was administered if CCRT was chosen as the initial treatment [25,26]. Patients receiving only systemic chemotherapy or target therapy were excluded. The choice of first-line treatment was based on the decision of the attending physician. The findings of all examinations in each individual and the pre-treatment staging were discussed and determined at a multidisciplinary lung cancer conference convened by our thoracic oncology research team.

### 2.2. Imaging Protocol and Analysis of ^18^F-FDG PET Scan

All study participants fasted for at least 4 h before ^18^F-FDG injection (400 MBq) and had blood glucose levels no greater than 200 mg/dL. The ^18^F-FDG PET/CT scans were performed 45 to 60 min after radiotracer administration using a GE Discovery ST scanner (GE Healthcare, Milwaukee, WI, USA). The PET/CT system was equipped with a PET unit containing 10,080 bismuth germanate crystals in 24 rings and a 16-detector row transmission CT unit. CT scans were performed first for attenuation correction without administration of contrast medium. The voltage and current of the tube were 120 kV and 120 mA, respectively. The pitch of the CT was 1.75 and sampling of CT images was done in the helical mode with a helical thickness of 3.75 mm. Immediately after the transmission CT, PET images were acquired from the midthigh to the vertex in a static 3-dimensional mode. The scanning time was three min for each table position (15 cm for each table position, with a 3 cm overlap for every contiguous frame). PET images were reconstructed with an ordered-subset expectation maximization algorithm (2 iterations, 21 subsets, and a 2.14 mm full width at half maximum Gaussian post-filter). The imaging matrix size, pixel size, and slice thickness for the reconstructed PET images were 128 × 128, 5.47 × 5.47, and 3.27 mm, respectively.

The platform used for display and semiquantitative analysis of ^18^F-FDG PET/CT images was a PMOD 4.0 system (PMOD Technologies Ltd., Zurich, Switzerland). An experienced nuclear medicine physician interpreted the ^18^F-FDG PET images. For image quantification, the experienced nuclear medicine physician identified and placed the volume-of-interest (VOI) on the primary tumor and the regional metastatic nodes on the ^18^F-FDG PET/CT image. The VOIs were placed and segmented separately for the primary tumor and the metastatic nodes. The SUV of ^18^F-FDG PET was calculated and normalized to each patient’s body weight as follows:

$$\mathrm{SUV}\;=\frac{\left(\mathrm{decay}-\mathrm{corrected}\;\mathrm{activity}\;\left(\mathrm{kBq}\right)\;\mathrm{per}\;\mathrm{milliliter}\;\mathrm{of}\;\mathrm{tissue}\;\mathrm{volume}\right)}{\left(\mathrm{injected}\;18F-\mathrm{FDG}\;\mathrm{activity}\;\left(\mathrm{kBq}\right)/\mathrm{body}\;\mathrm{weight}\;\mathrm{in}\;g\right)}$$

The ^18^F-FDG lesions were segmented using a 41% threshold of the maximum standard uptake value method [27]. The segmented volumes were used to define the MTV. The PMOD 4.0 software automatically generated the mean SUV within the MTV. The TLG was then calculated as TLV = mean SUV × MTV. The VOI and segmentation results were confirmed by another expert nuclear medicine physician. We recorded the SUV_max_ and TLG values of the primary tumors (described as primary tumor SUV_max_ and primary tumor TLG) and the regional metastatic nodes (nodal SUV_max_ and nodal TLG). Furthermore, we calculated the nodal to primary tumor SUV_max_ ratio (NTSUVR), the nodal to primary tumor TLG ratio (NTTLGR), the product of the primary tumor and nodal SUV_max_ (TNSUVproduct), and the sum of the primary tumor and nodal TLG (total TLG) based on the following formulas:

$$\mathrm{NTSUVR}\;=\frac{\left(\mathrm{nodal}\;\mathrm{SUVmax}\right)}{\left(\mathrm{primary}\;\mathrm{tumor}\;\mathrm{SUVmax}\right)}$$

$$\mathrm{NTTLGR}\;=\frac{\left(\mathrm{nodal}\;\mathrm{TLG}\right)}{\left(\mathrm{primary}\;\mathrm{tumor}\;\mathrm{TLG}\right)}$$

TNSUVproduct =(primary tumor SUVmax)×(nodal SUVmax)

total TLG = primary tumor TLG + nodal TLG

**Figure 1:**
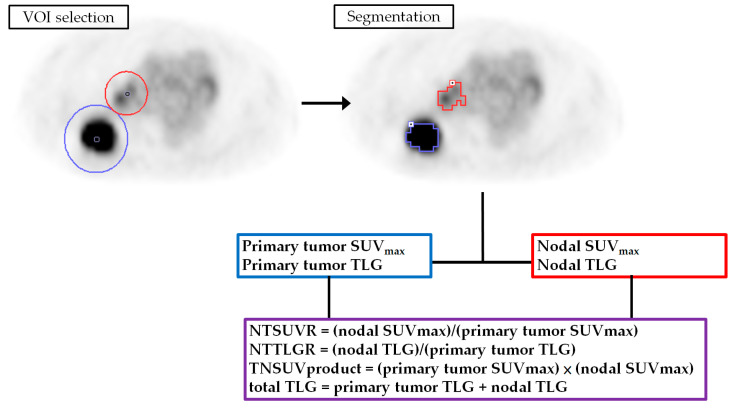
The method used for feature extraction from ^18^F-FDG PET. VOI, volume-of-interest; SUV, standardized uptake value; TLG, total lesion glycolysis; NTSUVR, nodal to primary tumor SUV_max_ ratio; NTTLGR, nodal to primary tumor TLG ratio; TNSUVproduct, product of primary tumor and nodal SUV_max_.

The procedure used for image feature extraction is outlined in Figure 1.

### 2.3. Follow-Up of Study Participants

After diagnosis, we followed patients with weekly outpatient clinic visits during treatment, at 3-month intervals after initial curative treatment, at 6-month intervals for 2 years, and annually thereafter. When signs of disease recurrence or progression emerged, contrast-enhanced CT, gadolinium-enhanced MRI of the brain, or ^18^F-FDG PET/CT were performed. Biopsies were taken for suspicious lesions whenever possible. New bloody effusion or positive fluid cytology was considered as recurrence or disease progression.

### 2.4. Data Analysis

We followed all study participants until death or March 2021 (whichever occurred first). Patient demographics were expressed as frequencies (percentage), means (standard deviation), or medians (interquartile range). The primary endpoints were OS and progression-free survival (PFS). The OS was defined as the date of cancer diagnosis to the date of death or censored at the date of the last follow-up for surviving patients. PFS was calculated from the date of treatment initiation to the date of disease progression (e.g., growth of a residual tumor or development of new metastatic lesion), the date of disease recurrence after complete remission, the date of death, or censoring at the date of the last follow-up. Continuous variables were selected and the optimal cut-off values for each continuous variable were determined using receiver-operating characteristic (ROC) curve analyses. Only variables that were statistically significant predictors of death in the ROC curve analyses were selected for the survival analysis. Cut-off values with the highest summation of sensitivity and specificity were selected as the optimal cut-offs for each continuous variable [28,29,30]. The variable selection and optimal cut-off determinations are summarized in the Supplementary Table S1. The continuous variables adjusted in the survival analysis were age, primary tumor SUV_max_, primary tumor TLG, nodal SUV_max_, nodal TLG, total TLG, and TNSUVproduct, and their optimal cut-off values were 75.5, 8.05, 42.5, 2.94, 18.3, 81, and 27, respectively. We used univariate and multivariate Cox regression analyses to study the association of the study variables with survival outcomes. First, we tested the effects of each variable on OS and PFS using univariate analyses. Then, the statistically significant variables from the univariate analysis were incorporated into the multivariate analysis to identify independent predictors of survival. We expressed the results of the survival analysis as hazard ratios (HR) and 95% confidence intervals. Statistical analyses were performed using SPSS software (version 20.0; SPSS Inc., Chicago, IL, USA).

The results of the multivariate Cox regression analysis were used to model the OS and PFS. The patient survival hazard was calculated by multiplying the HRs of the existing independent risk factors. For example, if a patient had three independent risk factors (HR1–HR3), the total hazard for this patient was calculated as HR1 × HR2 × HR3. If a patient had no risk factors, then the total hazard was the baseline hazard. If a patient had risk factors 1 and 3, then the risk of having shorter survival compared to no risk factor was HR1 × HR3.

### 2.5. Survival Model Validation and Comparison

The results of the multivariate Cox regression survival analysis were validated using the bootstrapping method. The validation process was performed with 1000 bootstrap samples. The results of bootstrapping validation were expressed as β, bias-corrected accelerated 95% confidence intervals, standard errors, and *p*-values. The validation process was executed using SPSS software (version 20.0; SPSS Inc., Chicago, IL, USA). The performance of our Cox regression models was assessed and compared with the AJCC staging system using Harrell’s concordance index (c-index) and Kaplan–Meier curves (log-rank test) [19,31]. Different c-indices were compared using the “compareC” package installed on the R open-source statistical software version 3.4.2 (R Foundation, Vienna, Austria). A two-tailed *p*-value of <0.05 was considered statistically significant.

## 3. Results

### 3.1. Patient Characteristics

Eighty-nine patients were eligible for analysis, whose baseline characteristics are summarized in Table 1. In total, 15 (16.9%), 53 (59.6%), and 21 (23.6%) patients were clinically classified as N1, N2, and N3 status, respectively. Thirty-seven (41.6%) patients received curative surgery or neoadjuvant CCRT and surgery as the initial treatment. Fifty-two (58.4%) patients received definitive CCRT or definitive radiotherapy. The median (interquartile range, IQR) time from the ^18^F-FDG PET/CT to initiation of treatment was 14 (12) days. The median follow-up period was 25.4 months (range, 1.7–130.2 months) for all patients and 48.7 months (range, 10.0–130.2 months) for the 34 surviving patients. Sixty (67.4%) patients experienced recurrence or disease progression after or during the initial treatment; 32 of these patients had locoregional recurrence or progression only and 15 patients developed distant metastases without locoregional failure. The remaining 13 patients had both locoregional failure and distant metastasis. By the time of the last follow-up, 55 (61.8%) patients died. The 5-year OS and PFS rates were 33.4% and 24.7%, respectively.

### 3.2. Univariate and Multivariate Survival Analyses

Our ROC curve analysis identified six semiquantitative ^18^F-FDG PET parameters that were associated with patient death. These parameters were included in the survival analysis (Supplementary Table S1), including the primary tumor SUV_max_, primary tumor TLG, nodal SUV_max_, nodal TLG, total TLG, and TNSUVproduct. The median OS and median PFS were 31.5 months (range, 1.7–130.2 months) and 15.1 months (range, 0.2–126.8 months), respectively. The results of the univariate and multivariate Cox regression analyses are outlined in Table 2. The univariate analysis of OS showed that age > 75.5 year-old, squamous cell pathology, T2–T4 disease, ECOG status > 0, never received surgery, only received radiotherapy, primary tumor SUV_max_ > 8.05, primary tumor TLG > 42.5, nodal SUV_max_ > 2.94, nodal TLG > 18.3, total TLG > 81, and TNSUVproduct > 27 were associated with shorter OS. The univariate Cox regression analysis for PFS showed that age >75.5 year-old, never received surgery, only received radiotherapy, primary tumor SUV_max_ > 8.05, primary tumor TLG > 42.5, total TLG > 81, and NSUVproduct > 27 were predictive of shorter time to progression. The statistically significant variables in the univariate analysis were fitted into multivariate Cox regression models. Age > 75.5 year-old, ECOG status > 0, and total TLG > 81 independently predicted unfavorable OS, whereas age > 75.5 year-old and total TLG > 81 were independent risk factors for shorter PFS.

### 3.3. Survival Model Construction and Validation

The independent risk factors were used to develop prediction models for OS (age > 75.5 years, ECOG > 0, and total TLG > 81) and PFS (age > 75.5 years and total TLG > 81). The construction of our survival models is demonstrated in the Supplementary Figure S1. The risk for each patient was calculated by multiplying the hazard ratio of each risk factor. In the OS model, the HRs for age > 75.5 years, ECOG > 0, and total TLG > 81 were 2.6, 3.3, and 5.1, respectively; in the PFS model, the HRs for age > 75.5 years and total TLG > 81 were 2.7 and 3.3, respectively (Table 2). If a patient had all three independent risk factors for OS, the total hazard of poor OS for this patient was 43.8 (2.6 × 3.3 × 5.1). If a patient had no risk factor, then the total hazard was the baseline hazard. If a patient was > 75.5 years and had a total TLG > 81, then the risks of having a shorter OS and poor PFS compared to no risk factor would be 13.3 (2.6 × 5.1) and 8.9 (2.7 × 3.3), respectively. The resulting hazards ranged from 1 to 43.8 for the OS model and 1 to 8.9 for the PFS model. Patients with similar 5 year survival outcomes in the Kaplan–Meier curve analysis were re-stratified into one risk group. Finally, we obtained three separate risk categories (HR < 3, HR = 3–10, and HR > 10 for the OS model; HR < 3, HR = 3–5, and HR > 5 for the PFS model).

The bootstrap method was used to validate our survival analysis results. Supplementary Table S2 presents the results of the bootstrap validation. The β estimate of each independent prognosticator was statistically significant in predicting OS and PFS.

### 3.4. Model Performance and Comparison to AJCC Staging System

**Figure 2:**
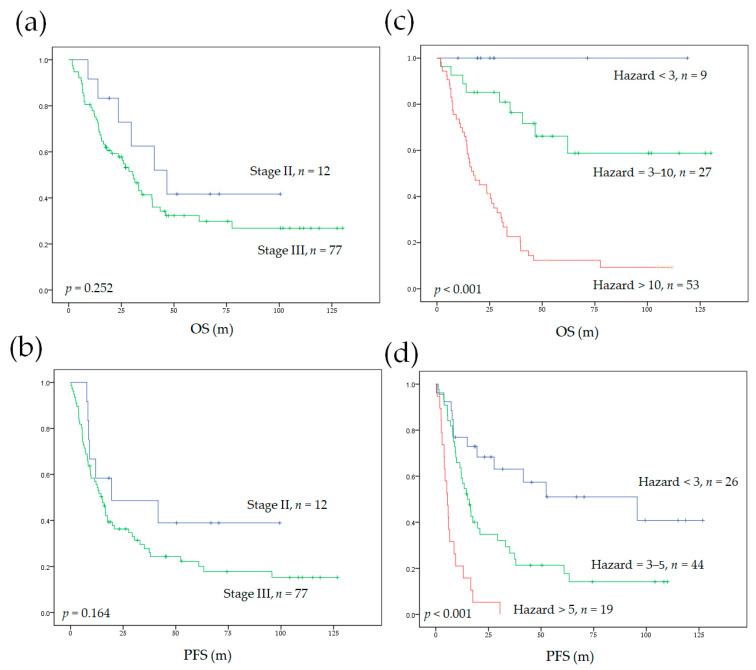
The Kaplan–Meier curves for OS and PFS in patients with nodal metastatic NSCLC without distant metastasis. Survival was stratified according to the eighth edition of the AJCC system (**a**,**b**) and our survival prediction model (**c**,**d**). OS, overall survival; PFS, progression-free survival; AJCC, American Joint Committee on Cancer.

The Cox regression models in our study significantly stratified patients into different survival risk groups (Figure 2). The c-indices of our Cox regression model for OS and PFS were 0.732 and 0.672, respectively. The Cox regression model developed in our study cohort significantly outperformed the AJCC staging system. In addition, our models (combining the independent clinical prognosticators with total TLG) showed the highest c-indices compared with other models using the combination of independent clinical risk factors with primary tumor TLG or nodal TLG (Table 3).

### 3.5. Model Performance in Subgroups of Different Initial Treatments

**Figure 3:**
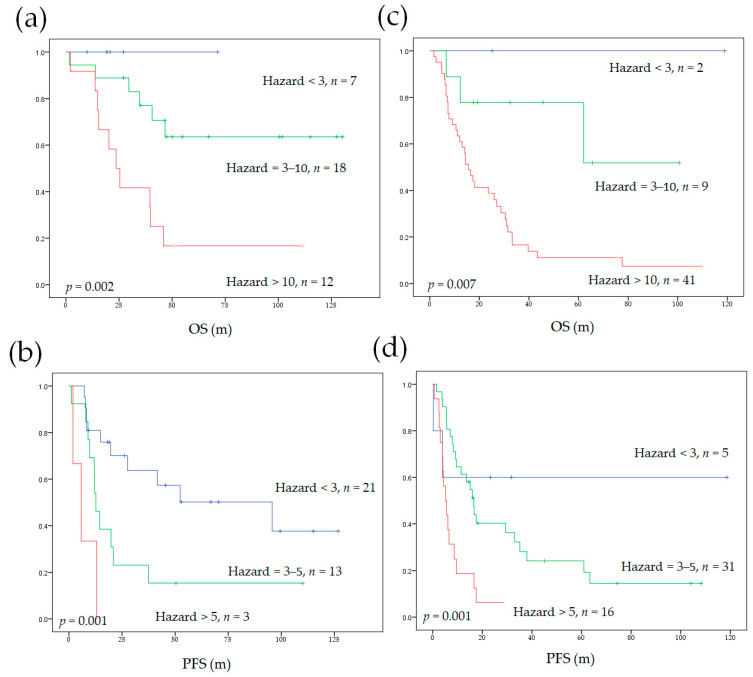
The Kaplan–Meier curves depicting OS and PFS stratified by our prediction model in subgroups of different initial treatments. The use of our model in subgroups that underwent curative surgery or neoadjuvant CCRT followed by surgery (**a**,**b**) and in initial non-surgical subgroup (**c**,**d**). OS, overall survival; PFS, progression-free survival; CCRT, concurrent chemoradiotherapy.

We also applied our survival prediction models to subgroups according to different initial treatments (curative surgery or definitive CCRT). Our models significantly stratified patients into different survival risks independent of the initial treatment strategy (Figure 3). The c-indices of our models were compared to the AJCC staging system in the subgroups. Our models significantly outperformed the AJCC staging system, except the c-index for PFS in patients who received initial curative surgery, which only showed a statistical trend (Table 4).

## 4. Discussion

Regional lymph node metastasis is common in NSCLC patients without distant metastasis (M0 disease) and is associated with a worse survival prognosis [32]. Disease recurrence within 5 years after initial curative treatment occurs in over half of lung cancer patients with nodal metastasis, and these patients eventually die of recurrence [9,10,32]. The survival outcomes of M0 patients with regional nodal metastatic NSCLC vary widely, despite initial aggressive treatment [8,9,11]. Therefore, a more reliable prognostic stratification tool is an unmet need. The prognostic value of ^18^F-FDG PET parameters derived from the primary tumor or the regional lymph node has been reported in patients with NSCLC [18,19,20,33,34]. However, the predictive power of combining the ^18^F-FDG PET parameters from both primary tumor and metastatic nodes has not been well investigated. Although some studies have combined the ^18^F-FDG PET volumetric parameters from the primary tumor and metastatic lesions, these study cohorts mixed locoregional disease cases with distant metastatic cases [35,36,37,38]. Thus, the results of these studies cannot be applied to patients with locoregional disease due to the diverse prognoses of patients with M0 or M1 diseases. In this study, we demonstrated that the total TLG derived from ^18^F-FDG PET, a combination of TLGs from both the primary tumor and regional lymph nodes, is an independent risk factor for PFS and OS in patients of locoregional NSCLC. Incorporating the total TLG with traditional clinical risk factors improved survival stratification.

Although the AJCC staging system is currently the mainstay of decision making regarding treatment strategies in NSCLC, it does not simultaneously assess tumor biological activity and burden. In patients with regional nodal metastatic NSCLC without distant spreading, the ^18^F-FDG PET metabolic parameters derived from primary tumor or metastatic nodes have been shown to be associated with survival outcomes in previous reports [18,33]. Because the primary tumor and metastatic nodes usually show different glucose metabolic profiles in ^18^F-FDG PET images, a combination of the two may provide comprehensive biological information for predicting prognosis. In this study, we found that combining the TLG of the primary tumor and the metastatic nodes into a total TLG resulted in an independent risk factor with a higher prognostic significance. The TLG is calculated by multiplying the MTV by the mean SUV, which weights the volumetric burden and metabolic activity of tumors. Kim et al. and Park et al. showed that larger primary tumor MTV was associated with a higher likelihood of occult nodal metastases [39,40]. Accordingly, larger metabolic tumor burden in patients with nodal metastatic NSCLC may also be expected to bear a higher risk of occult metastases in the more remote lymph node stations or even in distant organs. These occult lesions may escape from the most intensive treatment in the locoregional area. In addition to describing the tumor burden per se, the TLG also depicts the viability and the glycolytic activity of the tumor. The glycolytic pathway elicits diverse non-glycolytic mechanisms related to the promotion of cancer survival, proliferation, invasiveness, and adaptation to therapeutic agents, which are associated with unfavorable prognoses in patients with cancer [41,42]. Therefore, being a surrogate marker for both disease burden and vicious tumor behavior, total TLG may facilitate the stratification of patients into different risk groups.

Older age (>75.5 years) was an independent prognosticator for poor OS and PFS in our study. ECOG was also a prognostic factor for OS. Age and performance status are associated with survival outcomes in lung cancer as well as other malignancies, such as aerodigestive tract and gynecologic cancers [10,43,44,45,46]. The aged population has more medical comorbidities. In addition, aged patients may experience more toxicities from anti-neoplastic agents and suffer more perioperative complications, which may increase treatment-related mortality [47]. Moreover, the function of the T-cell-mediated immune system declines with age and limits the cellular immune response against tumor cells in the elderly population, further explaining the unfavorable survival outcomes in patients with advanced age [48,49,50,51]. We also analyzed the effects of different histopathological types on survival. Similar to other reports, squamous cell pathology was associated with worse survival outcomes compared with outcomes in patients with other histopathological types in the univariate analysis, whereas no statistical significance was found in the multivariate Cox regression analysis [20]. The histopathological type of NSCLC may vary according to age [9,46], in line with the age distribution in our cohort (Supplementary Table S3). Thus, the survival differences according to histopathologic types in our study appear to depend on patient age. Nevertheless, whether histopathological types impact the outcome of nodal metastatic NSCLC requires a more uniform patient cohort for verification.

**Figure 4:**
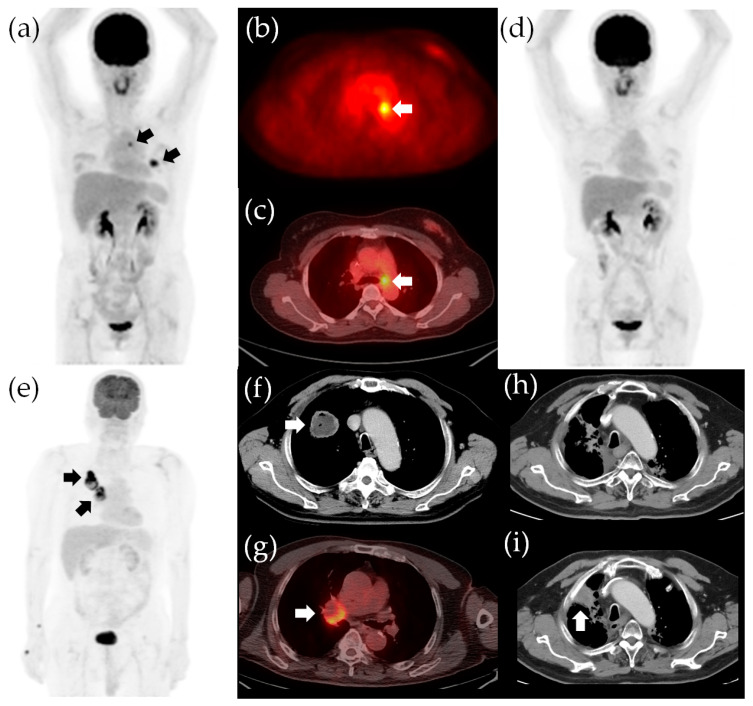
Survival stratification according to the independent risk factors in our study. The ^18^F-FDG PET/CT images for a 50-year-old woman with adenocarcinoma in the left upper lobe and subaortic nodal metastasis, indicated by arrows in the panels (**a**–**c**). The clinical staging was cT2aN2M0, stage IIIA. The total TLG was 14.9 and the ECOG status was 0. The patient had no poor survival risk factor (hazards were both 1 for an unfavorable OS and poor PFS) and she underwent lobectomy of the left upper lobe and mediastinal lymph node dissection. The pathological staging was pT2aN2M0, stage IIIA. She underwent adjuvant chemotherapy and is now alive without recurrence (**d**). The OS and PFS were 116 and 115 months, respectively. A 75-year-old man with adenocarcinoma in the right lower lobe and ipsilateral hilar nodal metastasis, indicated by arrows in the panels (**e**–**g**). The clinical staging was cT2bN1M0, stage IIB. The total TLG was 246.6 (>81) and the ECOG status was 1 (the hazards for unfavorable OS and poor PFS were 16.8 and 3.3, respectively). The patient received definitive CCRT (2 Gy/fraction daily to a targeted dose of 66 Gy) and marked tumor shrinkage was observed (**h**). However, the patient experienced progression of the primary tumor 15.9 months after definitive CCRT, indicated by the arrow in the panel (**i**). The patient eventually died of lung cancer progression, with an OS of 24.0 months and PFS of 15.9 months. TLG, total lesion glycolysis; OS, overall survival; PFS, progression-free survival; CCRT, concurrent chemoradiotherapy.

In our study, the total TLG is an independent risk factor depicting the disease, while the age and ECOG status characterize the host vitality. Survival outcomes in patients with cancer result from the complex interplay between the tumor and the host. Robust patient conditions with limited total TLG would have a higher chance of attaining disease-free status after curative treatment. On the other hand, vulnerable patient status and sizable total TLG are likely to experience treatment failure and eventually succumb to recurrence or disease progression (Figure 4). Therefore, incorporating both disease and host factors into one survival prediction model refines the prognostic stratification. Our survival stratification models also showed predictive value for survival outcomes in subgroups receiving different initial treatments. Because therapeutic decisions may vary from patient-to-patient based on clinical factors such as age, the baseline survival risk in subgroups receiving different initial treatment may vary as well [9,46]. For example, patients receiving curative surgery tend to be younger; thus, the surgical group has a lower baseline survival risk according to age. Nevertheless, the results of our study showed that our survival prediction model could be applied to different subgroups receiving different initial treatments, suggesting a wide utility of our survival stratification models in different treatment scenarios.

Despite the current therapeutic advances, treatment responses and survival times in nodal metastatic NSCLC patients are quite heterogeneous, and a reliable prognostic model for this patient group is still lacking [9,10,11]. For selected patients, salvage surgery for persistent or recurrent disease has been shown to improve disease control and may improve OS [52,53]. Furthermore, new therapeutic strategies have emerged that have improved disease control and prolonged survival in nodal metastatic NSCLC. For instance, adding adjuvant tyrosine kinase inhibitor in this patient group postpones recurrence in patients with an actionable epidermal growth factor receptor (EGFR) mutation [54]. Recently, preliminary data have suggested that neoadjuvant immunotherapy or chemoimmunotherapy may improve resectability and increase the pathological complete response rate [55,56,57,58]. However, sophisticated patient stratification before implementing these novel treatments is essential. Adding novel neoadjuvant or adjuvant therapy in patients with excellent survival outcomes after standard curative treatment may show little benefit and may result in undesirable adverse effects. Therefore, the survival prediction model in our study may aid in stratifying patients into different risk groups for tailored treatment decisions.

There were several limitations in our study. First, the patient cohort was not large and the study was conducted in a retrospective manner. In addition, heterogeneous patient characteristics such as the histopathological type of NSCLC and therapeutic strategies employed may introduce biases when analyzing survival data. Second, we did not include the EGFR mutation status in our survival analysis. Nonetheless, the meta-analysis by Zhang et al. showed that the EGFR mutation status was not predictive of the OS or disease-free survival in NSCLC with locoregional disease [59]. Furthermore, only 39 (43.8%) patients in our retrospective cohort were tested for EGFR mutation. Thus, we could not draw a clear conclusion on this issue. Finally, this study was performed in a single center and we only internally validated our results. Although external validation would be ideal before clinical implementation, external validation of a prognostic model requires a minimum of 100 events and ideally 200 events to produce reliable results [60]. Therefore, the generalizability of our survival prediction model warrants external validation in a larger prospective cohort.

## 5. Conclusions

Our preliminary results indicate that total TLG was a more significant independent prognostic factor than TLG when calculated from either primary tumor or metastatic nodes in predicting survival outcomes in patients with M0 NSCLC. Total TLG and age were predictive biomarkers for both OS and PFS, while ECOG status was an independent prognostic factor for OS. Combining total TLG with clinical factors yielded a survival stratification model that performed better than the traditional AJCC staging system. Our proposed survival stratification model may allow a more precise therapeutic approach in patients with nodal metastatic NSCLC without distant metastasis.

## Figures and Tables

**Table 1:** Baseline characteristics for patients in this study (*n* = 89).

Characteristics	Value
Age, years, mean ± SD	67 ± 11.4
Sex, n (%)	
Male	59 (66.3)
Female	30 (33.7)
Histology	
Adenocarcinoma	43 (48.3)
Squamous cell carcinoma	45 (50.6)
NSCLC—otherwise specified	1 (1.1)
T classification, n (%) ^a^	
T1b	2 (2.2)
T1c	16 (18.0)
T2a	10 (11.2)
T2b	11 (12.3)
T3	25 (28.1)
T4	25 (28.1)
N classification, n (%) ^a^	
N1	15 (16.9)
N2	53 (59.6)
N3	21 (23.6)
Overall stage, n (%) ^a^	
Stage IIB	12 (13.5)
Stage IIIA	23 (25.8)
Stage IIIB	41 (46.1)
Stage IIIC	13 (14.6)
ECOG, n (%)	
0	20 (22.5)
1	59 (66.3)
2	9 (10.1)
3	1 (1.1)
Initial treatment, n (%)	
Surgery	30 (33.7)
Neoadjuvant CCRT and surgery	7 (7.9)
Definitive CCRT	33 (37.0)
Definitive Radiotherapy	19 (21.4)
Time from ^18^F-FDG PET to initial treatment, d, median (IQR)	14 (12)
Quantitative analysis of ^18^F-FDG PET, mean ± SD	
Primary tumor SUV_max_	11.3 ± 5.22
Primary tumor TLG	292.1 ± 420.86
Nodal SUVmax	7.0 ± 4.96
NTSUVR	0.68 ± 0.415
Nodal TLG	67.4 ± 161.46
NTTLGR	0.79 ± 2.023
total TLG	359.6 ± 489.10
TNSUVproduct	89.7 ± 89.74

NSCLC, non-small cell lung cancer; ECOG, Eastern Cooperative Oncology Group; CCRT, concurrent chemoradiotherapy; SD, standard deviation; IQR, interquartile range; SUV, standardized uptake value; TLG, total lesion glycolysis; NTSUVR, nodal to primary tumor SUV_max_ ratio; NTTLGR, nodal to primary tumor TLG ratio; TNSUVproduct, product of primary tumor and nodal SUV_max_. ^a^ Staging according to 8th edition of American Joint Committee on Cancer manual.

**Table 2:** Univariate and multivariate analyses for survival prognostic factors.

Variable	No.	OS				PFS			
		Univariate		Multivariate		Univariate		Multivariate	
		HR (95% CI)	*p*-Value	HR (95% CI)	*p*-Value	HR (95% CI)	*p*-Value	HR (95% CI)	*p*-Value
Age			<0.001		0.001		0.001		<0.001
>75.5	24	2.8 (1.6–4.9)		2.6 (1.5–4.6)		2.5 (1.5–4.2)		2.7 (1.6–4.7)	
≤75.5	65	Reference		Reference		Reference		Reference	
Histopathology			0.012		0.126		0.175		NA
Squamous cell	45	2.0 (1.2–3.5)				1.4 (0.9–2.3)			
Others	44	Reference				Reference			
At least T2 disease			0.045		0.590		0.053		NA
Yes	71	2.3 (1.0–5.0)				2.0 (1.0–3.9)			
No	18	Reference				Reference			
N3 disease			0.303		NA		0.408		NA
Yes	21	1.4 (0.8–2.5)				1.3 (0.7–2.2)			
No	68	Reference				Reference			
Staging			0.257		NA		0.169		NA
Stage III	77	1.6 (0.7–3.8)				1.7 (0.8–3.8)			
Stage II	12	Reference				Reference			
ECOG status			0.007		0.022		0.084		NA
ECOG > 0	69	4.1 (1.5–11.4)		3.3 (1.2–9.4)		1.8 (0.9–3.6)			
ECOG = 0	20	Reference		Reference		Reference			
Received surgery ^a^			0.001		0.240		0.030		0.938
Absence	52	2.6 (1.5–4.7)				1.8 (1.1–2.9)			
Presence	37	Reference				Reference			
Radiotherapy only ^b^			<0.001		0.338		0.002		0.642
Yes	19	3.2 (1.8–5.6)				2.4 (1.4–4.2)			
No	70	Reference				Reference			
Primary tumor SUV_max_			<0.001		0.135		<0.001		0.238
>8.05	62	4.9 (2.2–10.9)				3.0 (1.6–5.6)			
≤8.05	27	Reference				Reference			
Primary tumor TLG			0.001		0.873		0.024		0.064
>42.5	63	3.7 (1.7–8.3)				2.0 (1.1–3.6)			
≤42.5	26	Reference				Reference			
Nodal SUVmax			0.012		0.114		0.204		NA
>2.94	70	3.0 (1.3–6.9)				1.5 (0.8–2.8)			
≤2.94	19	Reference				Reference			
Nodal TLG			0.014		0.454		0.104		NA
>18.3	40	2.0 (1.1–3.3)				1.5 (0.9–2.4)			
≤18.3	49	Reference				Reference			
total TLG			<0.001		<0.001		0.001		<0.001
>81	63	5.2 (2.2–12.2)		5.1 (2.2–12.0)		3.0 (1.6–5.7)		3.3 (1.7–6.2)	
≤81	26	Reference		Reference		Reference		Reference	
TNSUVproduct			<0.001		0.164		0.011		0.481
>27	67	5.3 (2.1–13.3)				2.3 (1.2–4.3)			
≤27	22	Reference				Reference			

OS, overall survival; PFS, progression-free survival; HR, hazard ratio; CI, confidence interval; ECOG, Eastern Cooperative Oncology Group; SUV, standardized uptake value; TLG, total lesion glycolysis; TNSUVproduct, product of primary tumor and nodal SUV_max_; NA, not applicable. ^a^ Received curative surgery or neoadjuvant chemoradiation followed by curative surgery. ^b^ Only received radiotherapy as the initial treatment.

**Table 3:** A Comparison of the c-indices between the traditional cancer staging system and our models.

Model	c-Index for OS	*p*-Value ^d^	c-Index for PFS	*p*-Value ^d^
AJCC staging system ^a^	0.544	NA	0.521	NA
Our Cox regression model	0.732	<0.001	0.672	<0.001
Model with primary tumor TLG ^b^	0.696	0.002	0.639	0.012
Model with nodal TLG ^c^	0.708	0.001	0.632	0.010

NA, not applicable; AJCC, American Joint Committee on Cancer; TLG, total lesion glycolysis. ^a^ Staging according to 8th edition of AJCC manual. ^b^ Model constructed from the independent clinical risk factors and primary tumor TLG. ^c^ Model constructed from the independent clinical risk factors and nodal TLG. ^d^ In comparison with AJCC staging system.

**Table 4:** A comparison of the c-indices of our model with the traditional cancer staging system in subgroups of different initial treatment strategies.

**Initial Surgery Group (*n* = 37) ^a^**				
**Model**	**c-Index for OS**	***p*-Value**	**c-Index for PFS**	***p*-value**
Our Cox regression model	0.742	NA	0.657	NA
AJCC staging system ^b^	0.513	<0.001	0.531	0.074
**Initial non-surgery group (*n* = 52)**				
**Model**	**c-index for OS**	***p*-value**	**c-index for PFS**	***p*-Value**
Our Cox regression model	0.667	NA	0.627	NA
AJCC staging system ^b^	0.466	0.004	0.441	0.003

NA, not applicable; AJCC, American Joint Committee on Cancer. ^a^ Including initial curative surgery or neoadjuvant chemoradiation followed by curative surgery. ^b^ Staging according to 8th edition of AJCC manual.

## Data Availability

The data presented in this study are available on request from the corresponding author. The data are not publicly available due to privacy and ethical restrictions.

## Supplementary Materials

The following are available online at https://www.mdpi.com/article/10.3390/diagnostics11061065/s1: Table S1: Results of receiver operating characteristic curve analysis. Figure S1. In the OS model, the combination of three independent risk factors resulted in eight different patient hazards (from 1 to 43.8). The combination of two independent risk factors of the PFS model resulted in four different patient hazards (from 1 to 8.9). We further re-stratified patients with similar 5-year survival outcomes in the Kaplan–Meier curve analysis into one risk category. Finally, we obtained three separate risk categories in our survival models. OS, overall survival; PFS, progression-free survival. Table S2: The results of the bootstrapping validation of our survival analysis. Table S3: The mean age according to the histopathology and treatment strategy.
