# Supplementary material for: Prognostic Value of Combing Primary Tumor and Nodal Glycolytic–Volumetric Parameters of 18F-FDG PET in Patients with Non-Small Cell Lung Cancer and Regional Lymph Node Metastasis
Source: Diagnostics (Basel). 2021 Jun 9;11(6):1065. doi: 10.3390/diagnostics11061065 (PMC8228685; doi:10.3390/diagnostics11061065)
Supplement: Supplementary file 1 [file diagnostics-11-01065-s001.zip › diagnostics-1253614-SI.pdf]

Supplementary Materials

Prognostic Value of Combing Primary Tumor and Nodal Glycolytic-Volumetric Parameters of ^{18}F-FDG PET in Patients with Non-Small Cell Lung Cancer and Regional Lymph Node Metastasis

Yu-Hung Chen [1,2], Sung-Chao Chu [2,3], Ling-Yi Wang [4], Tso-Fu Wang [2,3], Kun-Han Lue [5], Chih-Bin Lin [2,6], Bee-Song Chang [7], Dai-Wei Liu [2,8], Shu-Hsin Liu [1,5], Sheng-Chieh Chan [1,2*]

[1] Department of Nuclear Medicine, Hualien Tzu Chi Hospital, Buddhist Tzu Chi Medical Foundation, Hualien 97002, Taiwan; kaopectin@yahoo.com.tw (S.-H.L.); williamsm.tw@gmail.com (S.-C.C.); jedimasterchen@hotmail.com (Y.-H.C.)

[2] School of Medicine, College of Medicine, Tzu Chi University, Hualien 97002, Taiwan

[3] Department of Hematology and Oncology, Hualien Tzu Chi Hospital, Buddhist Tzu Chi Medical Foundation, Hualien 97002, Taiwan; oldguy-chu1129@umail.hinet.net (S.-C.C.); tfwang@tzuchi.com.tw (T.-F.W.).

[4] Epidemiology and Biostatistics Consulting Center, Department of Medical Research, Hualien Tzu Chi Hospital, Buddhist Tzu Chi Medical Foundation and Department of Pharmacy, School of Medicine, Tzu Chi University, Hualien 97002, Taiwan; wangly1212@gmail.com

[5] Department of Medical Imaging and Radiological Sciences, Tzu Chi University of Science and Technology, Hualien 97005, Taiwan; john.lue@protonmail.com

[6] Department of Internal Medicine, Hualien Tzu Chi Hospital, Buddhist Tzu Chi Medical Foundation, Hualien 97002, Taiwan; ferlin@tzuchi.com.tw

[7] Department of Cardiothoracic Surgery, Hualien Tzu Chi Hospital, Buddhist Tzu Chi Medical Foundation, Hualien 97002, Taiwan; rr122336@gmail.com

[8] Department of Radiation Oncology, Hualien Tzu Chi Hospital, Buddhist Tzu Chi Medical Foundation, Hualien 97002, Taiwan; dwliu5177@yahoo.com.tw

* Correspondence: williamsm.tw@gmail.com (S.-C.C.); Tel.: +886-3-856-1825

Table S1. Results of Receiver-Operating Characteristic Curve Analysis. (n = 89, Using Death as the Event)

Variable	AUC	p-value	Cutoff	Sensitivity	Specificity
Age	0.630	0.040	75.5	36.4%	88.2%
Primary tumor SUV_{max}	0.731	< 0.001	8.05	87.3%	58.8%
Primary tumor TLG	0.765	< 0.001	42.5	87.3%	55.9%
Nodal SUV_{max}	0.662	0.011	2.94	89.1%	41.2%
NTSUVR	0.443	0.366	0.72	43.2%	45.0%
Nodal TLG	0.660	0.012	18.3	58.2%	73.5%
NTTLGR	0.389	0.080	0.03	79.5%	7.5%
total TLG	0.766	< 0.001	81	89.1%	58.8%
TNSUVproduct	0.736	< 0.001	27	92.7%	50.0%

Figure S1. In the OS model, the combination of three independent risk factors resulted in eight different patient hazards (from 1 to 43.8). The combination of two independent risk factors of the PFS model resulted in four different patient hazards (from 1 to 8.9). We further re-stratified patients with similar 5-year survival outcomes in the Kaplan-Meier curve analysis into one risk category. Finally, we obtained three separate risk categories in our survival models. OS, overall survival; PFS, progression-free survival.

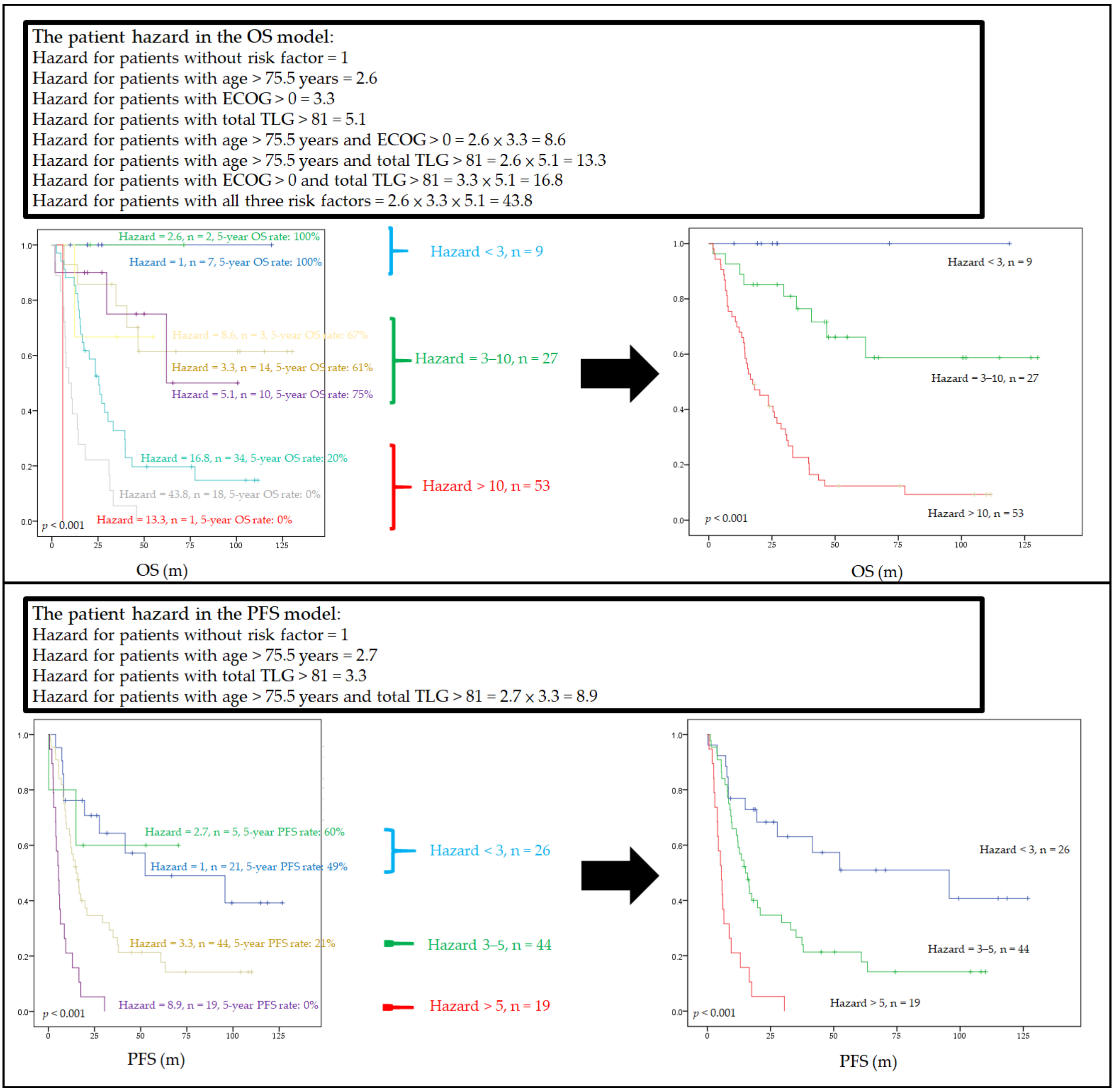

Table S2. The Results of the Bootstrapping Validation of our Survival Analysis.

		β (95% CI)	Bias	SE	p-value
OS	Age > 75.5	0.9 (0.3 – 1.8)	0.048	0.334	0.003
	ECOG > 0	1.2 (0.1 – 12.5)	0.271	1.458	0.019
	total TLG > 81	1.6 (0.7 – 3.3)	0.121	0.730	0.002
PFS	Age > 75.5	1.0 (0.4 – 1.7)	0.015	0.377	0.002
	total TLG > 81	1.2 (0.5 – 2.0)	0.036	0.286	0.002

OS: Overall survival, PFS: Progression-free survival, 95% CI: Bias corrected accelerated 95% confidence interval, SE: Standard error.

Table S3. The mean age according to the histopathology and treatment strategy.

Variables	Mean age (SD)	*p*-value
Histopathology		0.049
Squamous cell carcinoma	69 (10.6)	
Others	64 (11.8)	
Curative surgery		0.169
Yes	65 (10.2)	
No	68 (12.1)	
Radiotherapy only		< 0.001
Yes	78 (9.1)	
No	64 (10.2)	

SD, standard deviation.
